# Weight Loss Interventions and Skeletal Health in Persons with Diabetes

**DOI:** 10.1007/s11914-022-00744-9

**Published:** 2022-08-30

**Authors:** Qi Zhao, Sonal V. Khedkar, Karen C. Johnson

**Affiliations:** 1 Department of Preventive Medicine, College of Medicine, University of Tennessee Health Science Center, Memphis, TN 38163 USA; 2 College of Medicine, University of Tennessee Health Science Center, Memphis, 38163 TN USA

**Keywords:** Bone, Fracture, Lifestyle intervention, Type 2 diabetes, Weight loss

## Abstract

**Purpose of Review:**

Weight loss is recommended for improving glycemic control and reducing cardiovascular risk factors in persons with diabetes. However, both diabetes and weight loss have been associated with detrimental skeletal health. This review aims to summarize recent study findings on the effects of lifestyle interventions for weight loss on skeletal health among persons with type 2 diabetes (T2D).

**Recent Findings:**

A few large-scale observational studies have demonstrated an increased fragility fracture risk associated with weight loss among persons with T2D. Randomized control trials in persons with T2D also have shown that intentional lifestyle interventions for weight loss are associated with a greater decrease in bone mineral density (BMD) and an increase in the risk of fracture. The biological mechanisms underlying the compromised bone health during lifestyle interventions for weight loss are complex and not yet conclusive. However, there is evidence to suggest that bone loss and increased fracture risk during intentional weight loss may be mitigated by some intervention approaches, such as high protein intake, calcium supplementation, and resistance and balance training.

**Summary:**

There is still a lack of studies investigating the effects of different interventions for weight loss on skeletal health among persons with T2D. However, certain types of diet and physical activity intervention combined with bone monitoring and fracture risk prediction may help achieve weight loss goals and maintain skeletal health among persons with T2D during intentional weight loss.

## Introduction

Diabetes mellitus, especially type 2 diabetes (T2D), has emerged as a leading public health concern worldwide because of its escalating prevalence and considerable impact on human life and health expenditures [1, 2]. Diabetes was the ninth leading cause of death globally in 2019 and is a major cause of blindness, kidney failure, heart attacks, stroke, and lower limb amputation [3]. It was estimated that about 14.7% of the US adults (37.1 million) had diabetes in 2019, and approximately 90–95% of them had T2D [4]. The total estimated cost of diagnosed diabetes in 2017 was $327 billion, representing a 26% increase from 2012 [5]. Among the risk factors (e.g., obesity, physical inactivity, and a poor diet) [6], obesity has the closest relationship with T2D because both of them are associated with insulin resistance [7]. Nearly 90% of persons with T2D are overweight or obese [4]. Men and women with obesity are approximately 7 and 12 times more likely to develop T2D compared to those with normal weight, respectively [8]. Obesity also contributes to diabetes progression and cardiovascular disease. Therefore, weight loss is a critical component of managing diabetes and is recommended for most persons with T2D who are overweight or obese by the American Diabetes Association [9].

Although there are well-established beneficial effects of weight loss on improving cardiovascular risk factors in persons with T2D, weight loss has been associated with bone loss and potentially increased risk of fracture. It is also well-known that persons with T2D have an increased incidence of fracture despite having increased bone mineral density (BMD) [10]. Therefore, the potential negative impact of weight loss on skeletal health has become a concern for the treatment of T2D, and thoughtful considerations for preserving bone are necessary for weight loss planning. This review aimed to highlight the importance of weight loss in diabetes, the most recent study findings on skeletal health during weight loss interventions among persons with diabetes, and potential interventional approaches to preserve/improve bone health during weight loss. Because the pathophysiology of type 1 diabetes and T2D are different and obesity and weight loss are more relevant to T2D, we focused our discussion on T2D in this review. Additionally, weight loss caused by medications or bariatric surgeries is beyond the scope of this review.

## Weight Loss in Diabetes

The importance of weight management in T2D is well recognized. Both observational [11, 12] and clinical [13] trials have provided evidence that for persons with T2D, weight loss could significantly improve glycemic control, measured by both fasting plasma glucose and HbA1c, and other cardiovascular risk factors. The Look AHEAD (Action for Health in Diabetes) study, a multicenter randomized clinical trial, is still the largest long-term trial that was designed specifically to examine the effect of weight loss on cardiovascular outcomes in persons with T2D who are overweight or obese. Of 5145 persons enrolled at 16 centers across the USA, half were randomly assigned to receive an intensive lifestyle intervention (ILI) for weight loss and the other half to a diabetes support and education (DSE) group. ILI was designed to achieve and maintain a weight loss of at least 7% by facilitating reduced caloric intake and increased physical activity. Although Look AHEAD did not observe a protective effect of weight loss on cardiovascular morbidity and mortality over 9.6 years of follow-up, it did observe improvement in cardiovascular risk factors among the ILI group, such as decreases in HbA1c, systolic and diastolic blood pressure, triglycerides, and urine albumin-to-creatinine ratio and increase in HDL cholesterol and treadmill fitness [14, 15]. Additionally, a post hoc analysis of Look AHEAD suggested an inverse association between the magnitude of weight loss and the incidence of cardiovascular disease in persons with T2D [16]. Furthermore, Look AHEAD also found that complete or partial remission of diabetes was possible with weight loss in the ILI group [17]. Another two recent randomized clinical trials, DiRECT [18] and DIADEM-I [19•], also provide further evidence to support the potential remission of T2D by weight loss through lifestyle intervention.

## Skeletal Health in Diabetes

Persons with diabetes have shown an increased risk for bone fragility fracture compared with persons without diabetes. A large-scale meta-analysis including over 17 million participants from 49 studies concluded that both T1D and T2D were associated with a higher risk of hip and non-vertebral fractures. In T2D, insulin use and longer diabetes duration are associated with a greater risk of hip fractures [20]. The mechanism for the increase in the risk of fractures in diabetes is still not fully understood and might be associated with several features of T2D.

Unlike persons with T1D who typically have decreased BMD, persons with T2D usually have normal or elevated BMD as measured by dual-energy x-ray absorptiometry (DXA). So poor bone quality, such as deteriorated bone microarchitecture, has been suggested to contribute to skeletal fragility in T2D. Three major techniques [the trabecular bone score (TBS), high-resolution peripheral quantitative computed tomography (HRpQCT), and microindentation] have been used to evaluate bone microstructure in T2D [21]. TBS is derived from the two-dimensional lumbar spine DXA image, with a lower score indicating a reduced number of trabeculae and less connectivity. There is evidence to support that persons with T2D have a significantly lower TBS value despite higher BMD levels, suggesting TBS may be a useful approach for the diagnosis of bone fragility and evaluation of fracture risk in T2D [21]. However, the findings using the HR-pQCT are conflicting. For example, the Framingham Study reported that T2D had lower cortical volumetric BMD (vBMD), higher cortical porosity, and smaller cross-sectional area at the tibia, but not radius, compared with persons without T2D [22•]. Conversely, another study reported an increased ultradistal tibial and radial trabecular bone volume fraction, distal cortical vBMD, and cortical area in persons with T2D [23]. Microindentation is invasive, and existing studies are limited with relatively small sample sizes. A recent study showed that men with T2D had lower bone material strength index (BMSi) using the microindentation approach compared to men without T2D [24]. Therefore, further large-scale studies are still required to identify powerful indicators for bone fragility by these techniques to facilitate fracture risk prediction in T2D.

Another possible mechanism contributing to bone fragility in T2D is the accumulation of advanced glycation end-products (AGEs) resulting from prolonged hyperglycemia. AGEs accumulation leads to the generation of undesired crosslinks in bone, which affects mineralization, material properties, and microstructure, thereby impairing biomechanical properties and reducing bone strength [25•]. Persons with T2D have higher levels of AGE and nonenzymatic cross-link ratio and lower tissue-level (nanoindentation) modulus and hardness in the femoral head bone compared to those without T2D [26]. Elevated levels of AGE pentosidine and sugars bound to the collagen matrix were also observed in cancellous specimens of the femoral neck from persons with T2D [27]. Carboxy-methyl-lysine, a non-fluorescent AGE and less studied, has been associated with an increased risk for incident fracture in T2D, suggesting its potential implication in the pathogenesis of bone fragility in diabetes [25].

The increased risk for fracture is also related to the increased risk of falls in diabetes. Persons with T2D suffer a greater risk of falls [28, 29]. The common causes of falls in T2D include insulin use, microvascular complications (such as retinopathy, nephropathy, and neuropathy), and hypoglycaemic episodes [30, 31]. Sarcopenia also has been suggested as a potential risk factor for falls in T2D. However, few studies have investigated the associations between T2D and muscle tissue and the risks of falls and fractures [32]. Antidiabetic medications might also be involved in the increased risk of fracture in T2D [33, 34]. A meta-analysis found that thiazolidinediones (TZDs) and canagliflozin of the sodium-glucose cotransporter 2 (SGLT2) were associated with an increased risk of fracture, whereas metformin, sulfonylureas, insulin, dipeptidyl peptidase-4 (DPP-4) inhibitors, and glucagon-like peptide-1 (GLP-1) agonists appeared to have no effects on fractures [33].

## Weight Loss and Skeletal Health

Although it has been established that weight loss has multiple physiological and metabolic benefits, there is a substantial body of evidence that weight loss can lead to bone loss and an increased risk for fracture. Large-scale longitudinal cohort studies also observed an increased risk of fracture among individuals with intentional or unintentional weight loss [35–38]. Clinical trials involving older adults with obesity only have shown intentional weight loss without concomitant exercise training decreased total hip BMD [39]. A meta-analysis of clinical trials not limited to the aging population also found that diet-induced weight loss induced a decrease in total hip BMD [40]. In addition to decreased BMD, weight loss may also have detrimental effects on bone microarchitecture and strength. The Framingham Offspring Cohort has reported that long-term and recent weight loss was associated with lower cortical density and thickness, higher cortical porosity, and lower trabecular density and number at the tibia and radius measured by HR-pQCT [41].

## Weight Loss Interventions and Skeletal Health in Diabetes

Both diabetes and weight loss have negative impacts on bone health. However, weight loss is still recommended for diabetes patients who are overweight or obese for glycemic control and the prevention of comorbidities. There is still a lack of research exclusively conducted in persons with T2D to examine the effect of weight loss on bone health and fracture risk. Two recent observational studies reported weight loss was associated with increased fracture risk in persons with T2D [42, 43]. Komorita et al. used a diabetes registry data including 4706 persons with T2D (2755 men and 1951 postmenopausal women) and found the hazard ratios (HR) for fragility fracture in men were 2.23 (95% CI: 1.08–4.64) and 5.20 (95% CI: 2.15–12.57) for persons with 20–30% and ≥ 30% weight loss from maximum weight, respectively. The results showed the same trend in women but were not statistically significant [42]. Another large study included over 1.4 million subjects with T2D from medical insurance data and examined the association between weight change within 2 years and hip fracture risk. Both weight loss ≥ 10% and weight gain ≥ 10% were associated with an increased risk of hip fracture, and weight loss ≥ 10% showed a higher HR (1.61 vs. 1.46) [43].

Although these observational studies may provide further evidence of weight loss-related bone loss in persons with T2D, it was unclear if the weight loss experienced by the study subjects was intentional or unintentional. Look AHEAD is still the largest randomized clinical trial of long-term intentional weight loss through ILI among overweight/obese persons with T2D. Look AHEAD has observed a greater short-term and long-term decrease in BMD among the subjects of the ILI group compared with those of the DSE group. In a substudy of Look AHEAD which included 642 subjects of ILI and 632 of DSE from 5 of 16 clinical centers, BMD was measured using DXA at years 1, 4, 8, and 12–16 years after randomization. At 1-year follow-up, BMD was significantly decreased at the total hip and femoral neck in ILI but not for the lumbar spine and whole body [44]. At 4-year follow-up, in men, the bone loss rate was still greater in ILI than in DSE although the bone loss rate between the two groups was diminished. However, in women, no difference in the rate of bone loss between ILI and DSE was observed [45]. Even after 8 years and 12–16 years, the total hip BMD was still significantly lower in the ILI group compared to the DSE group in men but not in women, whereas more women in ILI had osteoporosis or low bone density at the lumbar spine compared with DSE (31.1% vs. 21.7%) [46]. In addition to bone loss induced by ILI, Look AHEAD did observe a 39% increased risk of frailty fracture in ILI, a composite of the first occurrence of a hip, pelvis, upper arm, or shoulder fracture, through a median of 11.3 years of follow-up [47•].

**Fig. 1:**
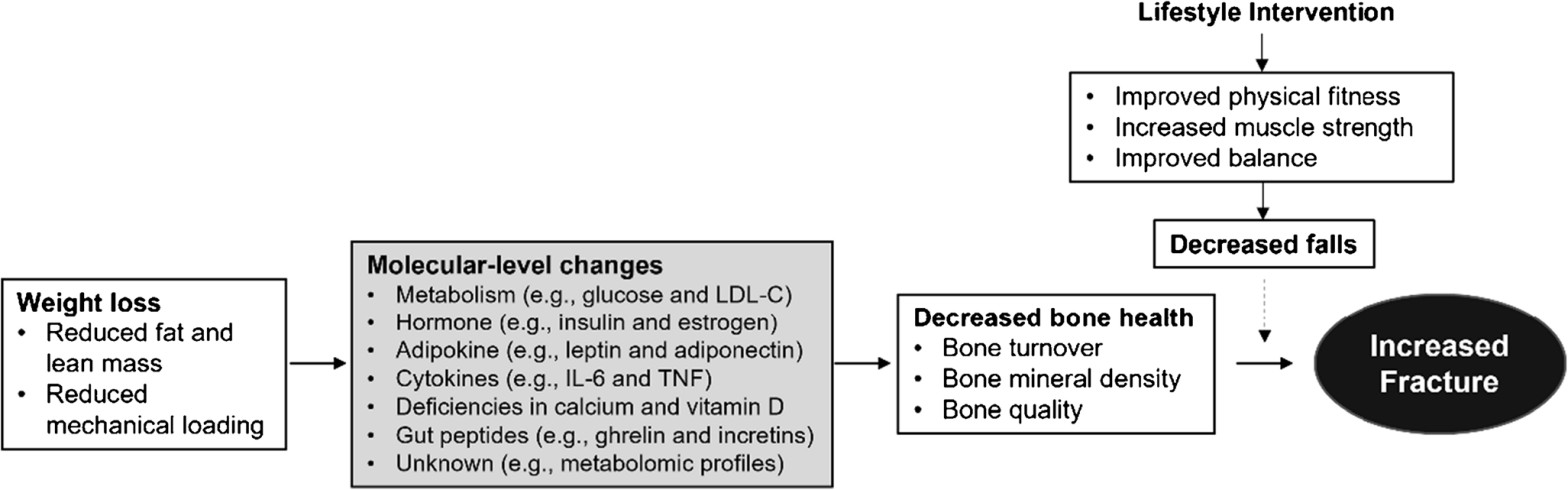
Possible mechanisms involved in the weight loss-induced increased risk of fracture in T2D

The biological mechanisms underlying bone loss and increased risk of fracture during intentional weight loss by lifestyle intervention among persons with T2D are complex and largely unknown (Fig. 1). As we discussed, both T2D and weight loss can have detrimental effects on bone and have been associated with an increased risk of fracture. The mechanisms underlying the impact of T2D on bone health are complex, some of which may have contradictory effects. For example, hyperglycemia in diabetes results in the accumulation of AGEs potentially leading to inferior bone quality, whereas hyperinsulinemia in T2D increases osteoblast proliferation and bone formation [48]. Weight loss can lead to reduced BMD through decreased mechanical loading, and weight loss-induced decreases in estrogen and other sex hormones also lead to a rise in bone resorption [49]. Changes in cytokines (e.g., IL-1, IL-6, and TNF-α), adipokines (e.g., leptin and adiponectin), calcium and vitamin D deficiency, and even gut-derived hormones may also play roles in bone metabolism change during weight loss [49, 50]. In addition to poor BMD and bone quality, falls also influence the risk of fracture. However, weight loss induced by lifestyle intervention usually involves improved physical fitness and body balance, likely leading to reduced falls [51]. In Look AHEAD, we did observe decreased falls in the ILI group compared to the DSE group (37.5% vs. 42.0%) [47•]. Therefore, the mechanisms underlying the increased fracture risk during weight loss in T2D might be more complicated than in those without T2D. Also, the contribution of each possible mechanism may vary by the different approaches used for weight loss. These warrant continuing mechanism research for providing effective methods to prevent fractures during weight loss in persons with T2D.

## Potential Approaches to Maintain Bone Health During Weight Loss Among Persons with T2D

It seems clear that weight loss can induce bone loss and subsequently increase the risk of fracture. Also, weight loss is still the recommended first-line treatment for T2D to improve glycemic control and possibly prevent cardiovascular disease and other comorbidities. Therefore, it may be necessary to monitor bone health and evaluate fracture risk before and during weight loss. Different intervention methods for weight loss may have different effects on bone [52]. It is important to optimize the methods of lifestyle intervention to reduce bone loss and the risk of fractures. Some recent advances in these fields were discussed.

### Bone Monitoring and Fracture Risk Prediction

It has been considered that the use of the Fracture Risk Assessment Tool (FRAX) underestimates fracture probability in individuals with T2D [53, 54]. Many FRAX-independent risk factors have been identified for the risk of fracture among persons with T2D, which may contribute to improving the risk prediction of fractures. For example, TBS from DXA which measures bone microarchitecture is independent of FRAX and may provide additional prediction value beyond BMD, especially for persons with T2D who usually have elevated BMD but damaged bone microstructure [55]. Weight loss is usually accompanied by lean mass loss. A large registry-based cohort study found that lean mass loss measured using DXA was associated with the incidence of major osteoporotic fracture and hip fracture independent of FRAX [56]. Height loss may also predict incident fracture independently from FRAX [57]. Furthermore, the precision of DXA in the setting of weight change and for individuals with body weight over 300 lbs can be compromised [58]. DXA may overestimate BMD with aging due to the development of osteophytes and other degenerative changes, particularly in the spinal vertebra. However, QCT has been thought to be superior to DXA at detecting bone loss and osteoporosis [59, 60]. More research is still needed to develop better tools for fracture risk prediction among persons with T2D, especially considering lean mass and even fat mass changes during weight loss. DXA and even QCT scans may be performed before and during weight loss interventions to actively monitor and evaluate bone changes and fracture risk. Proper treatments or other interventions should be applied once excessive fracture risk is detected.

### Diet Choice

Energy restriction combined with low fat or low carbohydrate intake is commonly used for weight loss [61, 62]. Recent research suggests that a higher protein diet can mitigate the loss of lean mass during intentional weight loss among older adults. Although the effect of protein intake and bone health is still not very clear [63], a recent randomized trial suggests a hypocaloric and high-protein meal plan could result in weight loss while maintaining similar bone density and quality to weight-stable controls at 6 months after interventions [64]. However, another study shows that even combined with high protein intake relative to total energy intake, severe energy restriction (65–75% of energy intake before intervention) still induced bone loss [65]. There is also an ongoing clinical trial to examine the effect of a 6-month high protein (30 g/meal) with caloric restriction intervention on muscle and bone health at 18 months [66].

There is evidence to suggest that bone loss with intentional weight loss may be mitigated by calcium supplementation [67]. However, vitamin D supplementation is still controversial for its protective effect on bone health. A recent clinical trial even observed high-dose vitamin D supplementation was negatively associated with radial and tibial BMD, with a greater adverse effect on BMD among females compared to males [68, 69]. There are very limited studies investigating the effect of vitamin D supplementation during weight loss on bone health. A small randomized controlled trial including 58 older women did not see any beneficial effect of vitamin D supplementation on BMD and bone quality measured by pQCT except for a decreased decline in cortical thickness of the tibia after a 1-year intervention for weight control [70]. Another trial did not see any changes in BMD by vitamin D supplementation during weight loss among 218 postmenopausal women with vitamin D insufficiency [71]. Well-designed and large-scale randomized clinical trials are still needed to clarify the effect of vitamin D supplementation on bone health during weight loss.

Besides what to eat, when to eat may influence weight as well. Time-restricted eating (TRE) has been shown effective for weight loss [72–74]. A small-scale clinical trial (*N*=20) has recently observed significant weight loss but no bone loss at 12 weeks of a self-selected daily 8-h eating window of ad libitum intake [75]. TRE may provide an alternative/complementary approach for weight loss with a potential for bone preservation. However, longer term well-designed studies with sufficient power are still needed to elucidate its applications (e.g., maximum weight loss) and long-term effects on bone in persons with T2D.

### Physical Activity

Two recent meta-analyses of randomized controlled trials concluded that exercise attenuates bone loss during weight loss induced by calorie restriction [76, 77]. Both studies reported the group assigned to exercise combined with calorie restriction had less decrease in femoral neck BMD but no difference in lumbar spine BMD compared with the group assigned to calorie restriction alone. However, a larger and later meta-analysis found only resistance exercise beneficially affected total BMD during a calorie-restricted diet [78]. This was further supported by a recent clinical trial which was not included in the meta-analyses above and examined the bone effects of aerobic (e.g., walking on a treadmill, stationary cycling, and stair climbing) and resistance exercise (e.g., flexibility exercise, resistance training using weightlifting machines, and balance exercise) during weight loss. This study found that compared with aerobic exercise, resistance and combined aerobic and resistance exercise decreased the loss in hip BMD and the increase in bone turnover during weight loss [79•]. A similar effect of resistance exercise on bone during weight loss was observed in people with T2D as well [80].

Preventing falls should be also considered for reducing the risk of fractures in persons with T2D who may have poor balance control and increased falls risk because of peripheral neuropathy. Balance training has improved balance and motor coordination and decreased the risk of falling in persons with T2D [81]. Therefore, resistance exercise combined with balance training may provide an effective approach for preserving bone and preventing falls and fractures during weight loss interventions.

There is still an ongoing effort to investigate novel methods to mitigate bone loss during intentional weight loss. For example, the Incorporating Nutrition, Vests, Education, and Strength (INVEST) study is investigating the effect of a novel weight replacement method (via weighted vest use) on bone during weight loss interventions [82•]. The method aims to maintain mechanical stress which decreases during weight loss and contributes to bone loss.

## Conclusions

Both T2D and weight loss have a deleterious impact on bone health. There are still very limited studies to investigate the approach to mitigate bone loss during weight loss interventions, especially for persons with T2D. Potential solutions may include optimization of diet and physical activity which minimize adverse bone effects during weight loss. The available evidence seems to support the potential application of calorie restriction combined with increased protein intake, resistance exercise, and balance training in weight loss interventions. Calcium plus vitamin D supplementation may also be beneficial. Also, novel biomarkers are required to improve the fracture risk prediction during weight loss to carry out timely treatment for the prevention of osteoporosis and fracture. In conclusion, future research is still warranted to investigate the approaches for maintaining and even improving bone health during weight loss and maximizing the health benefit from weight loss in persons with T2D.
